# A Novel Cadherin-like Protein Mediates Adherence to and Killing of Host Cells by the Parasite Trichomonas vaginalis

**DOI:** 10.1128/mBio.00720-19

**Published:** 2019-05-14

**Authors:** Yi-Pei Chen, Angelica M. Riestra, Anand Kumar Rai, Patricia J. Johnson

**Affiliations:** a Molecular Biology Institute, University of California, Los Angeles, California, USA; b Department of Microbiology, Immunology & Molecular Genetics, University of California, Los Angeles, California, USA; University of Pittsburgh

**Keywords:** Trichomonas vaginalis, host-pathogen interactions, parasite attachment

## Abstract

Trichomonas vaginalis, a prevalent sexually transmitted parasite, adheres to and induces cytolysis of human mucosal epithelial cells. We have characterized a hypothetical protein, TVAG_393390, with predicted tertiary structure similar to that of mammalian cadherin proteins involved in cell-cell adherence. TVAG_393390, renamed cadherin-like protein (CLP), contains a calcium-binding site at a position conserved in cadherins. CLP is surface localized, and its mRNA and protein levels are significantly upregulated upon parasite adherence to host cells. To test the roles of CLP and its calcium-binding dependency during host cell adherence, we first demonstrated that wild-type CLP (CLP) binds calcium with a high affinity, whereas the calcium-binding site mutant protein (CLP-mut) does not. CLP and CLP-mut constructs were then used to overexpress these proteins in T. vaginalis. Parasites overexpressing CLP have ∼3.5-fold greater adherence to host cells than wild-type parasites, and this increased adherence is ablated by mutating the calcium-binding site. Additionally, competition with recombinant CLP decreased parasite binding to host cells. We also found that overexpression of CLP induced parasite aggregation which was further enhanced in the presence of calcium, whereas CLP-mut overexpression did not affect aggregation. Lastly, parasites overexpressing wild-type CLP induced killing of host cells ∼2.35-fold, whereas parasites overexpressing CLP-mut did not have this effect. These analyses describe the first parasitic CLP and demonstrate a role for this protein in mediating parasite-parasite and host-parasite interactions. T. vaginalis CLP may represent convergent evolution of a parasite protein that is functionally similar to the mammalian cell adhesion protein cadherin, which contributes to parasite pathogenesis.

## Abstract (precis)

The adherence of pathogens to host cells is critical for colonization of the host and establishing infection. Here we identify a protein with no known function that is more abundant on the surface of parasites that are better at binding host cells. To interrogate a predicted function of this protein, we utilized bioinformatic protein prediction programs which allowed us to uncover the first cadherin-like protein (CLP) found in a parasite. Cadherin proteins are conserved metazoan proteins with central roles in cell-cell adhesion, development, and tissue structure maintenance. Functional characterization of this CLP from the unicellular parasite Trichomonas vaginalis demonstrated that the protein mediates both parasite-parasite and parasite-host adherence, which leads to an enhanced killing of host cells by T. vaginalis. Our findings demonstrate the presence of CLPs in unicellular pathogens and identify a new host cell binding protein family in a human-infective parasite.

## INTRODUCTION

Trichomonas vaginalis is an extracellular eukaryotic parasite that causes trichomoniasis, the most common nonviral sexually transmitted infection, which affects more than 275 million people worldwide annually (1). In the United States, trichomoniasis is classified as a neglected disease due to limited knowledge of the consequence of infection and its disproportionate affliction of low-income populations and minorities (2–4). Although the majority of T. vaginalis infections are asymptomatic, trichomoniasis can result in inflammation of the urogenital tract of both men and women, resulting in vaginitis, prostatitis, pruritus, dysuria, and discharge (5). Furthermore, T. vaginalis is also associated with adverse pregnancy outcomes and HIV coinfection (6–8).

As a parasite that does not invade host cells, it is critical for T. vaginalis to attach to urogenital epithelial cells in order to establish an infection and acquire nutrients from host cells (9). T. vaginalis strains also display different abilities to bind host cells *in vitro*, with up to a 45-fold difference in attachment observed between different strains (10). However, previous attempts to characterize specific parasite proteins involved in host binding suggested that the process is mediated by multiple factors, and many are yet to be defined (11–17). Therefore, understanding the molecular mechanisms of how T. vaginalis attaches to host cells is the key to understanding how the parasite establishes infection (13). In an attempt to determine what factors play a role in adherence, we previously compared the plasma membrane surface proteomes of 3 adherent and 3 less adherent T. vaginalis strains, identifying proteins that are significantly more abundant in the adherent strains than in the less adherent strains (17). Mining this surface proteomics data revealed a hypothetical protein, TVAG_393390, that is 1.7- to 3.4-fold more abundant in 2 out of 3 adherent strains than in less adherent strains (17). TVAG_393390 was also identified as a putative substrate of the T. vaginalis rhomboid protease 1 (TvROM1), a membrane serine protease that we have shown is involved in parasite attachment and host cytolysis (18). Together, these findings highlight a potential role for the TVAG_393390 protein in T. vaginalis-host cell interactions.

T. vaginalis cell-cell interactions may also be an important phenotype contributing to pathogenesis (11). Groups of parasites are readily visible when attached to ectocervical and prostate cells (15, 19). The ability of a T. vaginalis strain to aggregate also correlates with higher host cell adherence and cytolytic properties (20). In metazoans, the strongest forms of cell-cell attachment are mediated by cadherin proteins that bind to each other on apposing cells forming adherens junctions (21). While single-celled eukaryotes, such as T. vaginalis, do not contain cell junctions, it has been hypothesized that protein precursors in protozoans may have given rise to the complexes that allowed cell-cell interactions leading to multicellularity (22, 23). Bioinformatic analysis revealed that TVAG_393390 is predicted to be structurally similar to metazoan cadherin proteins, indicating its potential role in mediating cell-cell adhesion in the protozoan T. vaginalis.

Classic cadherin proteins are large single-pass transmembrane proteins with extracellular cadherin (EC) repeats, calcium-binding sites, and a cytosolic tail that is involved in intracellular signaling and interactions with p120 catenin, β-catenin, α-catenin, and, indirectly, F-actin (24, 25). Cadherin proteins are involved in both homophilic and heterophilic interactions (26). Homophilic interactions occur when cadherin protein binds to the same type of cadherin protein in *trans* on another cell. A classic example is the mouse epithelial cadherin (E-cadherin), which mediates cell-cell interactions in epithelial cells (27–29). Heterophilic interactions involve cadherin proteins binding to a different type of protein on a different cell type (30). An example of a heterophilic interaction that has a role in pathogenesis is the binding of the human E-cadherin protein to a Listeria monocytogenes surface protein, an interaction which helps to mediate invasion of the bacteria (30).

Here we characterize TVAG_393390 and show that it is functionally similar to cadherin proteins. This cadherin-like protein (CLP) was also found to play significant roles in parasite attachment to and lysis of host cells, as well as parasite aggregation. CLP may thus represent an evolutionary relic of cadherin-like proteins. To our knowledge, this is the first report of a cadherin-like protein in protozoans contributing to host-pathogen and parasite-parasite interactions.

## RESULTS

### Bioinformatic analysis reveals that TVAG_393390 is structurally similar to cadherin proteins.

**FIG 1:**
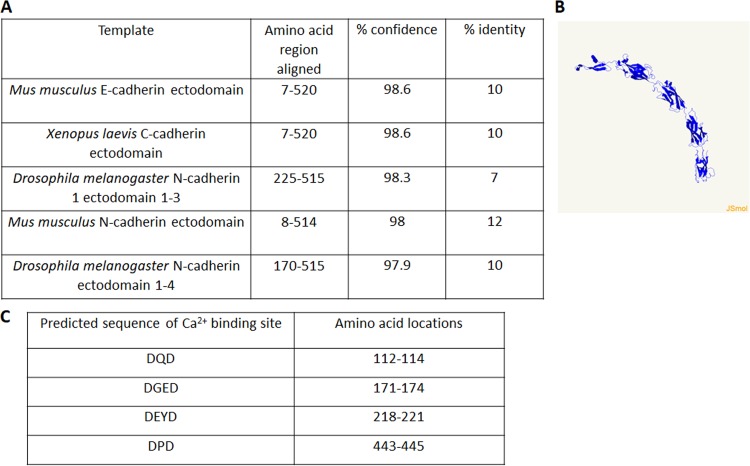
Tertiary structure modeling of TVAG_393390 predicts cadherin-like protein (CLP) function. (A) The most common high-quality 3D models of CLP predicted by Phyre2 revealed homology modeling to cadherin proteins. Characteristics of the aligned regions of these models are shown. (B) The predicted structure of TVAG_393390 generated by Phyre2 using one of the highest-confidence models, mouse E-cadherin, as the template. (C) Inspection of TVAG_393390 sequence for LDRE, DXD, and DXXD (X is any amino acid) revealed four predicted Ca^2+^-binding sites.

The T. vaginalis protein TVAG_393390 was found to be more abundant in the surface proteomes of adherent T. vaginalis strains than in less adherent strains, 1.7- to 3.4-fold (17). We also identified TVAG_393390 as a putative substrate of a T. vaginalis rhomboid protease demonstrated to be involved in parasite attachment and host cytolysis (18). The function of this protein is unknown, as it is annotated as a conserved hypothetical protein in the genome database for T. vaginalis (https://trichdb.org/trichdb/). To further investigate a potential role for TVAG_393390 in T. vaginalis adherence, we blasted both the gene sequence and the protein sequence using National Center for Biotechnology Information (NCBI) Basic Local Alignment Search Tool (BLAST) and found no homologues in other organisms. We also used InterPro (31) and Pfam (32) analyses to search for functional domains; none were identified. Next we employed a secondary structure prediction program called Phyre2, which predicts protein structure by comparing a large database of known protein secondary structures and building three-dimensional models based on the identified homologues (33). Using this approach, we found that the most common modeling for TVAG_393390 was to cadherin proteins, with each model having at least 97.9% confidence scores (probability that a template is homologous to our sequence) and 7% aligned residues (Fig. 1A). Figure 1B shows a TVAG_393390 model derived using one of the highest-ranking tertiary structures, mouse E-cadherin protein. Similar to cadherin proteins, TVAG_393390 is predicted to have 5 extracellular domains with the classical β-sandwich domains and Greek key folding of cadherin proteins (34, 35) (Fig. 1B). Additional bioinformatics analyses of TVAG_393390 with PredictProtein (36) (Fig. S1) and I-TASSER (37) (Fig. S2) further supported the predicted cell surface localization of TVAG_393390, calcium-binding sites, and structural similarity to cadherin family proteins. Due to TVAG_393390’s high-confidence modeling to cadherin proteins and the presence of key cadherin-like features, we renamed TVAG_393390 cadherin-like protein (CLP).

10.1128/mBio.00720-19.1 FIG S1 PredictProtein results for CLP. The PredictProtein server was queried with the CLP sequence. The table shows the protein hits that aligned to CLP. Note that the majority of proteins were T. vaginalis proteins. Two non-T. vaginalis proteins with high expected values for alignment are highlighted in yellow. The predicted cellular location of CLP at the plasma membrane as well as the highly sensitive mutational effects on the 4th predicted DPD (red-boxed region) calcium-binding site are also shown. Download FIG S1, TIF file, 4.1 MB. Copyright © 2019 Chen et al. 2019 Chen et al. This content is distributed under the terms of the Creative Commons Attribution 4.0 International license.

10.1128/mBio.00720-19.2 FIG S2 I-TASSER structural and ligand modeling results of CLP. (A) Results of the top 5 models and top 10 threading templates identified by I-TASSER for CLP. I-TASSER generates 5 top protein structural prediction models (bottom) using a metaserver threading approach called LOMETS. LOMETS utilizes multiple threading programs to produce threading alignments from Protein Data Bank (PDB) files. The top 10 best templates identified and ranked by I-TASSER used to predict the 5 top models are shown. Threading alignments that have a normalized Z-score of >1 are considered good alignments. Ident1 shows the percent sequence identity between the query and the threading program template. Ident2 is the percent sequence identity of the query compared with all the template chains. The percent coverage in the threading alignment (number of aligned residues/query protein length) is also shown. Note that only the hits highlighted in yellow had a TVAG_393390 (CLP) alignment with a potential Ca^2+^-binding site (LDRE, DXD, or DXXD) in that template and that the highest Z-scoring alignment was to the mouse E-cadherin ectodomain. (B) CLP ligand prediction using I-TASSER. Based on the I-TASSER-predicted structure of CLP, I-TASSER predicted the ligand binding sites shown in the table utilizing the programs COFACTOR and COACH. The C-score indicates the confidence score of the predicted ligand-binding site; values range from 0 to 1 with increasing reliability of the prediction. The cluster size of templates used to generate the prediction and the top PDB hit are shown. Lig Name, identity of the ligand that is predicted to be bound by the protein (CA, calcium; IPT, isopropyl-1-beta-d-thiogalactosidase/1-(isopropylthio)-beta-galactopyranside; MG, magnesium; ZN, zinc). Download FIG S2, TIF file, 5.5 MB. Copyright © 2019 Chen et al. 2019 Chen et al. This content is distributed under the terms of the Creative Commons Attribution 4.0 International license.

Another defining characteristic of cadherin proteins is their binding to extracellular Ca^2+^ via calcium-binding pockets located in between the extracellular domains (38). Visual inspection of the TVAG_393390 protein sequence for the highly conserved Ca^2+^-binding sites LDRE, DXD, and DXXD (X is any amino acid) (34), located at the interfaces of the extracellular domains predicted by Phyre2, identified four candidate Ca^2+^-binding sites (Fig. 1C). TVAG_393390 is also predicted to have one transmembrane domain, near the C terminus of the protein, based on the transmembrane protein topology prediction software TMHMM (39). Thus, the predicted orientation of TVAG_393390 would result in the cadherin-like modeled region being exposed to the outside of the cell with a small C-terminal tail located intracellularly (Fig. 1B).

### CLP is expressed on the surface of the parasite.

**FIG 2:**
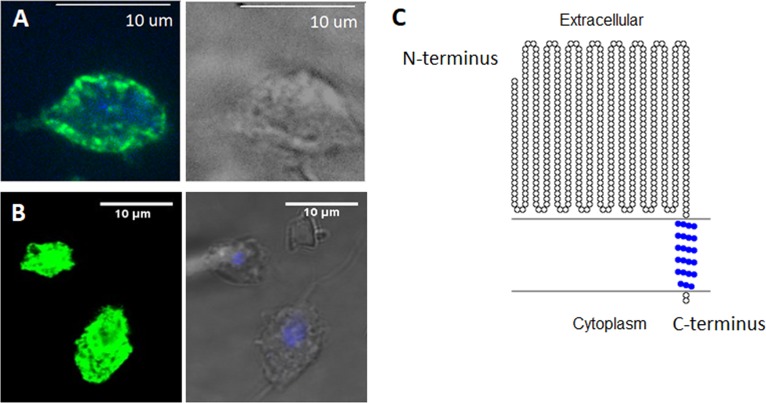
CLP is localized to the surface of the parasites. (A) Parasites exogenously expressing CLP with two C-terminal HA tags were stained for immunofluorescence microscopy using an anti-HA antibody (green) and 4′,6-diamidino-2-phenylindole (DAPI) for nuclear staining (blue). A bright-field image is shown on the right. (B) To further test CLP’s cell surface localization, CLP was exogenously expressed with an N-terminal GFP tag. Indirect immunofluorescence assays were performed using an anti-GFP antibody (green) without cell permeabilization. Merged images of DAPI staining and bright-field images are shown on the right. Indirect immunofluorescence images are representative of 60 parasites viewed in three independent experiments. (C) Predicted topology of CLP generated with the TOPO2 program. The predicted orientation is based on results from panels A and B and immunofluorescent assays performed in the absence or presence of a permeabilizing reagent (Fig. S1) as well as from the structural prediction from Fig. 1B. Predicted transmembrane residues are shown in blue.

To determine whether CLP is surface localized and displays the predicted orientation, we cloned the gene in our standard T. vaginalis expression vector, MasterNeo (40), under the control of a T. vaginalis α-succinyl coenzyme A (CoA) synthetase promoter and fused with two C-terminal hemagglutinin (HA) tags. The construct was then introduced into T. vaginalis by transfection, and parasites were selected with G418 as previously described (41). Using an indirect immunofluorescence assay with an anti-HA antibody, exogenously overexpressed CLP was found to localize to the surface of the parasite (Fig. 2A). Since it is also predicted that the C-terminal tail of the protein with the fused HA tags will be located inside the cell, to further probe the orientation of CLP, we performed indirect immunofluorescence with and without permeabilization. We found that the fluorescent signal from CLP is significantly stronger when the parasites are treated with a permeabilizing agent (Fig. S3A and C), as permeabilization allows anti-HA antibody access to the intracellular HA tags. To further test the protein localization and topology of CLP, we tagged CLP N terminally with green fluorescent protein (GFP) and performed indirect immunofluorescence assays in the absence of permeabilization. Using confocal microscopy, we additionally detected CLP cell surface localization (Fig. 2B). These results provided additional support for the notion that the C-terminal domain of CLP is located inside the cells. Our experimental results are consistent with the predicted CLP structure generated by Phyre2 analysis (Fig. 1B) and demonstrate that overexpression of the protein resulted in the predicted membrane localization with the cadherin-like domains being exposed on the outer surface of the parasite. The predicted CLP protein topology is graphically depicted in Fig. 2C.

### CLP mRNA and protein levels are upregulated during host contact.

**FIG 3:**
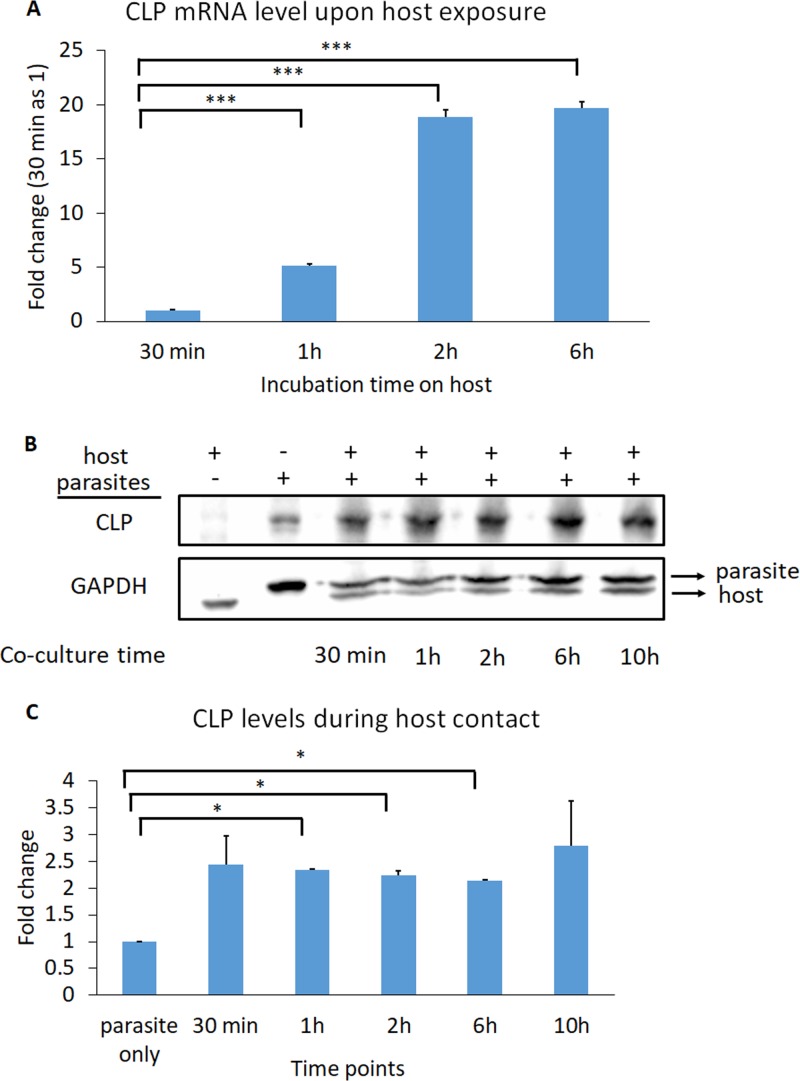
CLP levels are upregulated during host contact. (A) CLP mRNA is upregulated during host contact. T. vaginalis was exposed to host Ects for 30 min, 1 h, 2 h, and 6 h, and the amount of CLP mRNA was quantified by qRT-PCR. CLP mRNA levels relative to 30 min of exposure time are shown. CLP was upregulated 5-, 19-, and 20-fold after contact with Ects for 1 h, 2 h, and 6 h, respectively. A representative of 3 independent experiments is shown. Data shown are means of triplicates ± SDs. Statistical significance was determined using a one-way analysis of variance (ANOVA) with Dunnett’s multiple-comparison test. ***, *P* ≤ 0.001. (B) CLP is exogenously expressed driven by its own promoter and tagged with HA. The first lane is host only, and the second lane is parasites only. The parasites were incubated with the host for 30 min, 1 h, 2 h, 6 h, and 10 h (lanes 3 to 7). GAPDH was used as the loading control, with the upper band being the parasite GAPDH and the lower one being the host GAPDH. (C) Quantification of CLP levels was done by normalizing CLP signals to parasite GAPDH. The parasite-only control is set as 1. CLP levels were increased 2- to 3.5-fold during host contact. Data shown are means of triplicates ± SDs. Statistical significance was determined using one-way ANOVA with Dunnett’s multiple-comparison test. *, *P* ≤ 0.05.

Upon parasite contact with human ectocervical cells (Ects), T. vaginalis upregulates expression of a variety of proteins, including actin, actin-binding proteins (42), and tetraspanin proteins (TvTSP3, TvTSP5, TvTSP6, and TvTSP8), the last of which are transmembrane proteins that modulate their own expression and subcellular localizations during host contact (20, 43). Thus, we hypothesized that surface-localized CLP might be upregulated upon interaction of T. vaginalis with host cells. To test this, wild-type (WT) parasites were incubated with Ects for 30 min, 1 h, 2 h, or 6 h, followed by removal of unbound parasites in suspension and extraction of RNA from only the parasites adhered to Ects. We found that CLP mRNA was upregulated ∼5.2-fold, 18.9-fold, and 19.7-fold at 1 h, 2 h, and 6 h, respectively, relative to 30 min after host contact (Fig. 3A). As reduced temperature might affect the ability of the parasites to sense the environment initially and modulate surface protein expression, we used 30 min instead of 0 min for baseline comparison because immediately before coculturing the parasite with Ects, the parasites are exposed to icing to collect and concentrate them. To further test the role of host cell sensing in upregulating CLP levels, we expressed HA-tagged CLP driven by its own promoter and exposed these transfectants to Ects. We found that CLP levels were increased 2- to 3.5-fold upon contact with host cells (Fig. 3B and C). Interestingly, we also found that a pan-cadherin antibody that recognizes cadherins expressed by the Ects also bound to proteins on the parasites, and this signal was also increased after parasite contact with Ects (Fig. S5). This result may indicate that T. vaginalis expresses proteins structurally similar to cadherins, although future work is necessary to identify the potential CLPs recognized by the pan-cadherin antibody. Overall, these data support a role for CLP in host cell sensing and triggering upregulated expression.

10.1128/mBio.00720-19.3 FIG S3 Differential CLP staining. To determine the topology of overexpressed CLP, an indirect immunofluorescence assay in the presence or absence of a permeabilizing agent on C-terminally HA-tagged CLP was performed. (A and C) Bright green signal from anti-HA staining on permeabilized parasites (A) versus faint green in nonpermeabilized parasites (C) suggests that C-terminally tagged HA is on the intracellular side of the parasites. (B and D) Bright-field images of panels A and C, respectively. Green, HA; blue, DAPI. These images are representative of ∼30 parasites viewed under each condition. Download FIG S3, TIF file, 4.5 MB. Copyright © 2019 Chen et al. 2019 Chen et al. This content is distributed under the terms of the Creative Commons Attribution 4.0 International license.

### Calcium binding is predicted to be important for the function of CLP.

**FIG 4:**
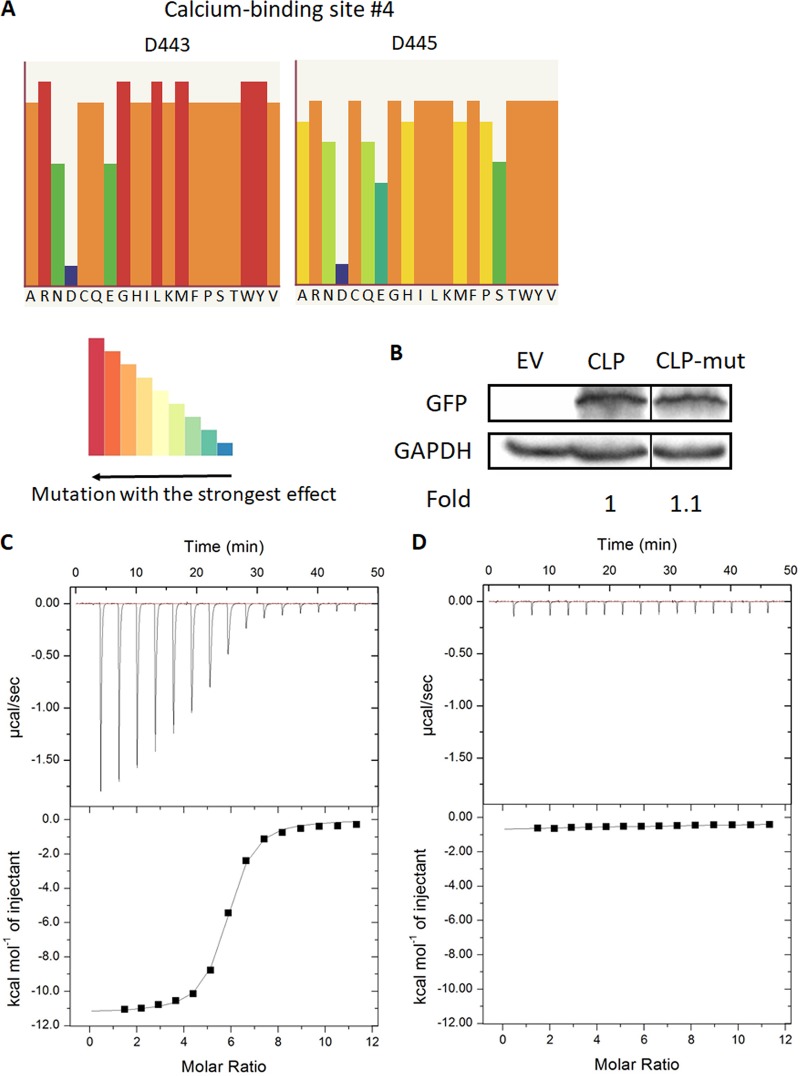
Generation of a CLP calcium-binding mutant and measurement of calcium interaction with WT and mutant CLP by isothermal titration calorimetry (ITC). (A) Phyre2 and SuSPect analyses identified the predicted calcium-binding site composed of D443 and D445 as the most sensitive to mutation (see Fig. S2 for analysis and comparison of other predicted calcium-binding sites in CLP). The height and color of the bars shown in the color key indicate the predicted functional impact of mutating the aspartate residue to the amino acids shown at the bottom of the histogram. Long red bars in the histogram indicate that introduction of that particular amino acid would lead to the greatest phenotypic change, while short blue bars have the smallest predicted phenotypic effect. (B) A CLP mutant that has D443 and D445 mutated to alanines (CLP-mut) was generated to investigate the functional effects of calcium binding in CLP. Wild-type CLP and CLP-mut were exogenously expressed with an N-terminal GFP tag. As a negative control, parasites were transfected with an empty vector plasmid (EV). Immunoblotting using an anti-GFP antibody confirmed that there were approximately equal amounts of wild-type CLP and CLP-mut overexpression. GAPDH is shown as a loading control. Fold represents the CLP expression levels between CLP and CLP-mut relative to CLP (=1). The thin black line between CLP and CLP-mut indicates the blot was spliced to remove an empty lane between the samples. (C and D) Results of using ITC to measure the binding of calcium to WT rCLP (C) and mutant rCLP (D). Calcium chloride (1 mM) was added to 14 μM WT rCLP or 14 μM mutant rCLP with 15 injections, 2 μl each, at 25°C. (C) The upper panel shows the heat change per second during the injection of calcium chloride to WT rCLP, and the changes decrease as the binding saturates. The lower panel shows the integrated heat changes after subtracting the heat generated from dilution. The binding *K_d_* for the WT rCLP was determined to be 0.729 μM. (D) The raw data (upper panel) suggest that there was no detectable calcium binding for mutant rCLP, and the estimated mutant *K_d_* was 1.236 mM.

Calcium binding plays a critical part in the adhesive role of cadherin proteins by rigidifying the extracellular domains and mediating binding between cadherin proteins on apposing cells (44, 45). Specifically, aspartate residues that help coordinate calcium ions at the base of the cadherin extracellular domains constitute one of the most conserved domains of cadherin proteins across different species (34). Therefore, to understand how CLP functions, we used Phyre2 and SuSPect mutational analysis to identify which aspartate residues of the 4 predicted Ca^2+^-binding domains (33, 46) (shown in Fig. 1C) would lead to the strongest phenotypic effects if mutated (Fig. 4A and Fig. S4). The two aspartate residues D443 and D445 in the putative fourth Ca^2+^-binding domain were predicted to be the most sensitive to mutation (Fig. 4A and Fig. S1 and S4), so we proceeded to mutate both of these residues to alanine, as this type of mutation is standardly performed to study the function of calcium-binding domains in cadherin proteins (47). We then overexpressed both WT CLP and the D443A D445A CLP mutant (CLP-mut) with an N-terminal GFP tag in T. vaginalis and an empty vector (EV) as a negative control. Next we compared the expression of CLP and CLP-mut in the selected transfectants and found that expression of WT CLP was similar to that of CLP-mut, as determined by immunoblotting using an anti-GFP antibody (Fig. 4B).

### Thermodynamics of binding between calcium and WT or mutant CLP.

To confirm calcium interaction with CLP empirically, we employed an isothermal titration calorimeter (ITC) approach which allows measurement of heat changes associated with biomolecular binding. We tested the interaction with calcium by titrating calcium chloride onto WT or mutant CLP. The heat changes were integrated and fitted to obtain the entire set of calcium binding thermodynamic parameters. Injection of calcium chloride into the solution containing WT CLP produced strong heat changes. With progressive injections, the heat changes decreased as the calcium-binding sites of CLP became saturated, and after 30 min, only background signals were observed (Fig. 4C). The integrals of the heat changes were then fitted according to the predicted number of calcium-binding sites present on the proteins. One-site binding model for CLP was the best fit, showing WT CLP having affinity with calcium (*K_d_* [dissociation constant] = 0.729 μM). Conversely, mutant CLP displayed weak binding (*K_d_* = 1.236 mM). The mutation in CLP resulted in complete disruption of calcium interaction, as the mutant protein did not display heat release while titrated with calcium chloride (Fig. 4D). Together, our ITC-based analyses demonstrate a single calcium-binding site on CLP and a highly significant decrease in calcium binding by the mutated CLP.

### CLP contributes to increased host cell binding, which is dependent on the CLP calcium-binding domain.

**FIG 5:**
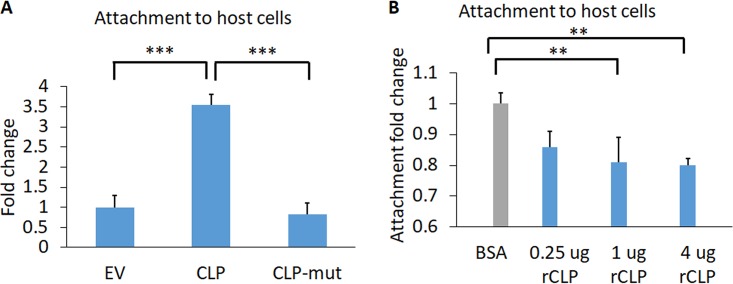
CLP contributes to T. vaginalis adherence to host Ects. (A) EV and CLP or CLP-mut transfectants were fluorescently labeled and incubated with Ects for 30 min, followed by quantification of adhered parasites. The average fold changes of CLP and CLP-mut relative to EV were 3.5- and 0.83-fold. Statistical significance was determined using one-way ANOVA with Tukey’s multiple-comparison test. (B) The ability of CLP’s extracellular domain to compete with T. vaginalis binding to Ects was tested by the addition of 0.25 μg (7.7 nM), 1 μg (30.8 nM), or 4 μg (123.2 nM) of rCLP. Exogenous rCLP decreased host binding by 14%, 19%, and 21% compared to that with the bovine serum albumin (BSA)-treated control. Statistical significance was determined using one-way ANOVA with Dunnett’s multiple-comparison test. Panels A and B represent 3 independent experiments, each performed in triplicate. **, *P* ≤ 0.01; ***, *P* ≤ 0.001.

To test whether CLP is important for host binding, we compared the abilities of CLP- and CLP-mut-overexpressing cells to bind host Ects. We found that overexpression of CLP increases the attachment to host cells 3.5-fold compared to the EV control (Fig. 5A). In contrast, parasites overexpressing CLP-mut have an attachment phenotype similar to that of EV (Fig. 5A). These data establish a role for CLP in host cell binding which is reliant on the presence of the calcium binding site.

To further investigate the role of CLP in host cell binding, we competed parasite binding to Ects with addition of recombinant CLP (rCLP). The extracellular domain (ECD) of rCLP was used instead of the entire protein for the ease of expressing the protein in Escherichia coli periplasm and since the ECD is predicted to be responsible for the cadherin protein-protein interactions (38). We added rCLP ECD to Ects for 30 min prior to the addition of parasites. We found that as the concentration of rCLP ECD was increased, the percentage of parasite binding to the host cell decreased (Fig. 5B). These results further support a contribution of CLP to host cell binding.

### CLP contributes to parasite-parasite clumping, and the effect is abolished in CLP-mut parasites.

**FIG 6:**
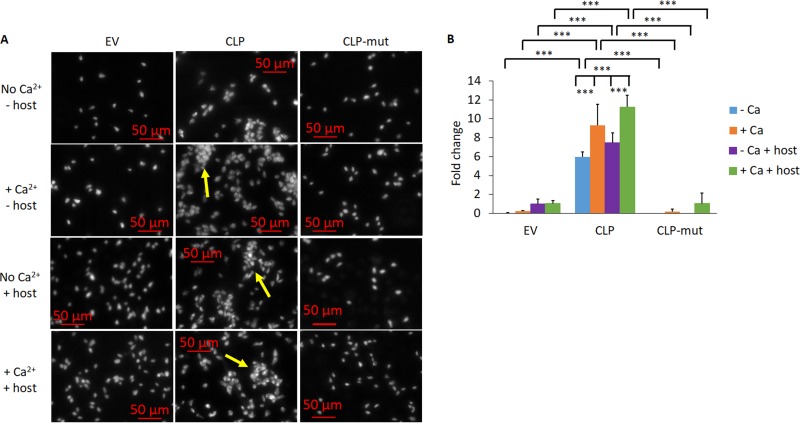
CLP increases parasite clumping, and CLP-mut reverses enhanced clumping. (A) The clumping ability of the parasites was assessed by quantifying an aggregate of 10 or more parasites. The parasites were incubated in the absence or presence of the host and in the absence or presence of 1 mM CaCl_2_. Images are at a magnification of ×100, and each white dot is a single parasite. Yellow arrows denote clumps with 10 or more parasites. (B) Quantification of clumping behavior observed in panel A. The fold changes shown are relative to the condition EV − Ca + host. The results represent those from 3 independent experiments, each performed in triplicate. Error bars indicate SDs. Statistical significance was determined using two-way ANOVA with Tukey’s multiple-comparison test. ***, *P* ≤ 0.001.

We and others have observed that more adherent T. vaginalis strains appear to clump with each other more readily than less adherent strains. In addition, overexpression of the TvTSP8 surface protein, which increases parasite adherence to host cells, also increased parasite clumping compared to that of the EV control parasites (10, 20). Furthermore, cadherin proteins are known to mediate homophilic interactions in which cadherin proteins interact with the same type of cadherin on another cell (26, 48, 49). We thus compared the clumping behavior of EV, CLP, and CLP-mut parasites with or without Ca^2+^ and in the presence or absence of the host cells. CLP displayed approximately a 7.5- to 240-fold increase in parasite clumping compared to the EV control (see Materials and Methods for the fold change calculations), and CLP-mut had almost the same levels of clumping as EV under all conditions tested (Fig. 6). Addition of Ca^2+^ but not the presence of host cells significantly increased the clumping of CLP parasites (Fig. 6). These data together suggest that the CLP-mediated clumping phenotype is calcium dependent and the ability does not necessitate a host signal.

### CLP contributes to increased killing of host cells.

**FIG 7:**
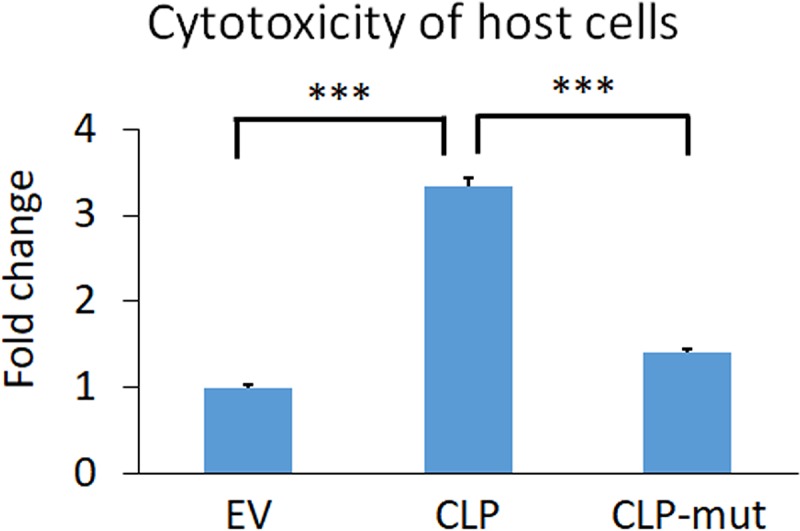
Death of Ects induced by T. vaginalis was increased by wild-type CLP overexpression 3.3-fold relative to the EV parasites, and the CLP-mut parasites reduced the cytotoxicity level to 1.4-fold that of the EV parasites. Data shown are means of triplicates ± SDs and 1 representative set of 3 independent experiments. Statistical significance was determined with one-way ANOVA with Tukey’s multiple-comparison test. ***, *P* ≤ 0.001.

Since CLP is involved in host cell binding and parasite clumping, we hypothesized that these properties could also contribute to increasing host cell death, as epithelial host cell killing by T. vaginalis is contact dependent (10) and the increased parasite-parasite association may further increase the number of parasites attacking the host. We found that CLP overexpression increased host death 3.3-fold compared to that with EV (Fig. 7). In contrast, CLP-mut displayed only a 1.4-fold increase in host killing compared to that with EV (Fig. 7). The slight increase in cytotoxicity observed with CLP-mut compared to that with EV may be due to the fact that although the majority of the protein depends on calcium binding for its function, CLP may make a calcium-independent contribution to host killing.

## DISCUSSION

We have identified and characterized a surface protein of T. vaginalis called cadherin-like protein (CLP) and showed that it plays a significant role in host binding, parasite clumping, and host cell killing. Mammalian cadherin proteins are known to be co-opted for pathogen invasion or colonization by several bacteria and the pathogenic yeast Candida albicans (50–52). However, the identification of CLPs or a role for unicellular pathogen CLPs in the adherence to and killing of mammalian cells has not been previously described, as this is the first functional analyses of a CLP in a unicellular eukaryote. We show that this T. vaginalis CLP mimics the structure and the function of host cadherin proteins, raising the possibility that other parasites may also use CLPs with low sequence identity but with structural similarity to mammalian cadherins to interact with host cells. The presence of a CLP in T. vaginalis that is structurally and functionally similar to mammalian cadherins is likely an example of convergence evolution of three-dimensional (3D) structures with similar properties, in the absence of strong primary sequence homology.

Cadherin proteins are known to be involved in homophilic interactions that result in adherence between cells of the same types (30, 48). We observed that CLP-overexpressing parasites clump (i.e., adhere to each other) significantly more than the empty vector control and the CLP mutant-overexpressing parasites, demonstrating a role for parasite CLP in homophilic adherence of parasites. We also demonstrated that CLP-induced parasite clumping is significantly increased in the presence of calcium, consistent with the calcium-dependent homophilic interactions mediated by cadherin proteins (48).

The effect of iron and zinc has been well documented to affect T. vaginalis gene expression, protease activity, and cytotoxicity (16, 53, 54). Here we expand the role of calcium ions contributing to T. vaginalis virulence functions. It is possible that the parasite integrates multiple sensory cues from metal and ion concentrations to drive its pathogenesis. We found that the potential Ca^2+^-binding site that was most strongly predicted to be functional by bioinformatic analyses was indeed responsible for CLP Ca^2+^ binding. ITC titrations revealed one binding site in CLP. Furthermore, we confirmed that the D443A D445A mutation in the potential Ca^2+^-binding domain of CLP abolishes the Ca^2+^ binding ability of the protein completely. The absence of Ca^2+^ binding by the mutant protein also suggested that the three other predicted binding sites either are not functional or display extremely low affinity toward Ca^2+^, as mutation in only one domain did not show any detectable Ca^2+^binding. Nevertheless, mutating the calcium-binding domain in CLP almost completely reversed the enhanced host attachment, parasite clumping, and host cell killing observed with parasites overexpressing WT CLP. It is possible that the overexpression of the T. vaginalis protein mutated in the calcium-binding domain results in a dominant negative effect on endogenous WT CLP. Future structural studies of CLP are therefore warranted to better understand areas of protein similarity and divergence with mammalian cadherins and how CLP biophysically interacts with itself and with host cell proteins, as well as how other ions may additionally contribute to CLP’s function or transcriptional upregulation.

Highly adherent strains of T. vaginalis have been observed to clump significantly more than poorly adherent strains (20); however, the role of clumping in infection is unclear. Another family of surface proteins, tetraspanins, have also been have been found to help mediate T. vaginalis aggregation (20). Interestingly, it has been reported that the human E-cadherin protein interacts with tetraspanin proteins in colon carcinoma (55). It is yet to be determined whether the T. vaginalis CLPs and TSPs functionally interact.

Preincubation of host cells with recombinant CLP (rCLP) reduced parasite binding to host cells, confirming a role for parasite CLP in host cell binding. While T. vaginalis attachment to host cells likely depends on multiple factors which have been identified (17, 18, 56–62), the largest amounts of rCLP tested reduced parasite binding by a significant 21% (Fig. 5B). Furthermore, the observed 3.5-fold increase in host binding is also among the strongest phenotypic effects mediated by exogenous expression of a single protein factor, compared to the 1.6-fold increase when rhomboid protease 1 is overexpressed and the 2.1-fold and 2.2-fold increases by TVAG_166850 TVAG_244130, two proteins previously identified in our surface proteome (17, 18). E-cadherin and N-cadherin are expressed in the human male and female urogenital and reproductive tracts (63, 64), and we also confirmed expression in the ectocervical cell line utilized in our studies (Fig. S5). E-cadherin and N-cadherin are also found on spermatozoa (64), which T. vaginalis can also attach to and phagocytose (65). It is therefore of interest to investigate whether CLP has a conserved role in attaching to and lysing multiple cell types and how it may structurally mediate molecular mimicry. Although the cell surface attachment properties of CLP are similar to those of mammalian cadherins, the intracellular signaling mediated by CLP is likely to differ since CLP has a very short predicted C-terminal tail. In mammalian cadherins, the C-terminal tail mediates signal transduction events via catenins (25); therefore, future work is necessary to uncover whether CLP associates with other proteins to mediate its function.

Parasite clumping effectively increases the number of parasites attaching to host cells, which, in turn, increases the likelihood of parasites successfully colonizing the host. Aggregation of parasites could also potentially protect parasites from immune destruction, as human neutrophils have been shown to kill T. vaginalis by taking bites from the parasites, i.e., via trogocytosis (66), and parasites in the center of an aggregate would be protected from neutrophil attack. Future analyses will better define the functional importance of parasites adhering to one another in parasite survival and infection.

Although CLP surface expression levels are different in adherent versus less adherent T. vaginalis strains, the fact that CLP is expressed by all the six strains surveyed in our prior proteomics study (17) may indicate an important and conserved function. Our work has uncovered new roles for a previously undescribed protein family in T. vaginalis, further elucidating mechanistic interactions contributing to pathogenesis. Our work also places T. vaginalis as a model eukaryotic microorganism for the broader study of cell-cell interactions and cell-cell adhesion.

## MATERIALS AND METHODS

### Bioinformatic analyses.

To predict TVAG_393390 function, we used InterPro (31), Pfam (32), Phyre2 (33), I-TASSER (37), and PredictProtein (36) programs to analyze its protein sequence and BLAST with both the gene and protein sequences published on TrichDB (67). For Phyre2 analysis, we chose the intensive modeling mode, then used the “Run Investigator” feature using the mouse E-cadherin template, and performed SuSPect mutational analysis on the four calcium-binding domains/eight aspartate residues in TVAG_393390 (46). The topology of TVAG_393390 was generated with the TOPO2 program (68).

### T. vaginalis and ectocervical cell line Ect1 culture.

T. vaginalis strain RU393 (ATCC 50142) was grown as previously described (69). Parasites were grown at 37°C and subcultured daily for up to 2 weeks. The human ectocervical cell line Ect1 E6/E7 (ATCC CRL-2614) was grown and passaged as previously described (14).

### CLP WT and mutant plasmid construction and T. vaginalis transfection.

TVAG_393390 (CLP) WT sequence was cloned into the MasterNeo-(HA)_2_ plasmid (40) or the N-terminal enhanced green fluorescent protein (eGFP)-MasterNeo plasmid (18). Two rounds of site-directed mutagenesis were performed using a QuikChange kit (Stratagene) in order to introduce the D443A and D445A mutations sequentially using the following primer sets. To introduce the D443A mutation, we used D443A Fwd (CACAGCCGTAGTTGTTGcTCCAGATACTAACTTTG) and D443A Rev (AAAGTTAGTATCTGGAgCAACAACTACGGCTGTG). To introduce the D445A mutation, we used D445A Fwd (GTAGTTGTTGcTCCAGcTACTAACTTTGATTCC) and D445A Rev (GGAATCAAAGTTAGTAgCTGGAgCAACAACTAC). Introduction of the desired mutations (indicated by lowercase letters in the primers) was confirmed by sequencing (Genewiz). The constructs were transfected into T. vaginalis strain RU393, and parasites containing the constructs were selected using G418 as previously described (41).

### Indirect immunofluorescence assays.

Parasite transfectants overexpressing WT CLP with two C-terminal HA tags were plated on glass coverslips coated with 100 μg/ml of poly-l-lysine (Sigma) and then fixed in 4% formaldehyde in phosphate-buffered saline (PBS) for 20 min. The cells were then permeabilized in 0.2% Triton X-100 in PBS or just PBS for a nonpermeabilized control for 15 min and blocked in 3% bovine serum albumin (BSA) for 30 min. A 1:1,000 dilution of anti-HA mouse (BioLegend) and 1:5,000 dilution of goat anti-mouse Alexa Fluor 488-conjugated secondary antibody (Molecular Probes) were used for staining. The coverslips were then mounted using ProLong gold antifade mountant with 4′,6-diamidino-2-pheylindole (DAPI; Thermo Fisher Scientific). The images were acquired using a Zeiss confocal microscope with a Yokogawa spinning disc and analyzed with SlideBook 6 software (Intelligent Imaging Innovations).

Anti-GFP indirect immunofluorescence assays were performed as described above except that noncoated glass coverslips and anti-GFP at a 1:1,000 dilution (Clontech) were utilized. Anti-cadherin indirect immunofluorescence assays were performed by growing Ects on glass coverslips overnight and exposing Ects to RU393 parasites for 4 h. Parasites were allowed to bind coverslips in parallel. A rabbit anti-pan-cadherin antibody (Abcam) was used at a 1:500 dilution and detected with a donkey anti-rabbit Alexa Fluor 594-conjugated secondary antibody (Life Technologies). Images were acquired on an Olympus FV100 confocal microscope and analyzed using Fiji software.

### Real-time quantitative reverse transcription-PCR (qRT-PCR).

A total of 1 × 10^7^ nontransfected RU393 parasites were incubated with 80% confluent Ects for 30 min, 1 h, 2 h, or 6 h. Unbound parasites were removed and RNA was collected by adding TRIzol to attached parasites on Ects. Total RNA was purified by phenol-chloroform extraction and treated with TURBO DNase (Invitrogen). cDNA was prepared by using SuperScript III with oligo(dT) primers (Thermo Fisher Scientific). Platinum SYBR green qPCR SuperMix-UDG and the manufacturer’s protocol (Thermo Fisher Scientific) were used for real-time PCR. T. vaginalis beta-tubulin was used as the housekeeping gene control. The primers for beta-tubulin were Tub-f (GGCTCGTAACACATCCTACTTC) and Tub-r (CTGTTGTGTTGCCGATGAATG). The primers for CLP were 393-f (GACGATGTTGTTAATTTCACAGCC) and 393-r (CCATCAGAGTTTGATCTTGAAATTGA). The specificity of the primers has been checked using NCBI Primer-BLAST, and the primers are unique to T. vaginalis sequences (70).

### Exogenous expression of TVAG_393390 driven by its own promoter to measure protein expression during host contact.

WT TVAG_393390 sequence in the MasterNeo-(HA)_2_ plasmid was used for cloning by swapping the α-succinyl CoA synthetase promoter to the endogenous TVAG_393390 promoter using primers 393_5′UTR-f (AATCATAACAAACAAGTAACAAAGACT) and 393_5′UTR-r (TACAAAGTGAAATGATTTTGATAAAGCC). The plasmid was transfected and maintained the same way as for WT and mutant CLP overexpression. A total of 5 × 10^6^ transfected parasites were incubated with 80% confluent Ects per well in 6-well plates. Parasite-only control was only the transfectant in a 6-well plate, and the host-only control was Ects in a 6-well plate. After 30 min, 1 h, 2 h, 6 h, or 10 h of host contact, unbound parasites were removed and the lysates were extracted with lysis buffer (0.1% Nonidet P-40, 0.5% deoxycholate, 2% SDS, 50 mM Tris [pH 8], 5 mM EDTA, 150 mM NaCl) plus 1× Halt protease inhibitor cocktail (Thermo Fisher Scientific) and incubated on ice for 30 min. The protein amount was determined by the bicinchoninic acid (BCA) protein assay (Thermo Fisher Scientific), and equal amounts were loaded from each sample for anti-HA immunoblot analysis to quantify CLP levels during host contact. The protein signals were quantified using Image Lab software (Bio-Rad) by normalizing CLP signals to glyceraldehyde-3-phosphate dehydrogenase (GAPDH) signals and setting parasite-only control as the reference.

### Immunoblot analyses.

Mouse anti-GFP polyclonal antibody (1:1,000; Clontech) and anti-GAPDH (1:10,000; Cocalico Biologicals) were used as the primary antibodies, and horseradish peroxidase (HRP)-conjugated anti-mouse (1:25,000) and anti-rabbit (1:25,000) antibodies (Jackson Laboratory) were used as the secondary antibodies. A total of 5 × 10^6^ parasites were taken from T. vaginalis culture and washed with PBS plus 5% sucrose plus 1× Halt protease inhibitor cocktail (Thermo Fisher Scientific). The cells were then lysed in lysis buffer (0.1% Nonidet P-40, 0.5% deoxycholate, 2% SDS, 50 mM Tris [pH 8], 5 mM EDTA, 150 mM NaCl) plus 1× Halt protease inhibitor cocktail (Thermo Fisher Scientific). The protein concentration was quantified using the BCA protein assay (Thermo Fisher Scientific), and equal amounts of protein were loaded from each sample. The protein signals were quantified using Image Lab software (Bio-Rad) by normalizing CLP signals to GAPDH signals and setting WT CLP as the reference.

### Production and purification of rCLP ECD.

Sequence encoding the CLP ECD was cloned into the pET22b(+) expression vector flanked by an N-terminal PelB periplasm signal sequence and C-terminal 6×His tag. The primers for cloning were pET22b_393-f (TAATTCGGATCCGATGATTTGGACTTTTTTATTGCAGGATG) and pET22b_393-r (ACTAAGCTCGAGCTTCTTTTGTTTTTGCTGCTTTCTTTG). The plasmid was transformed into E. coli C41(DE3) cells (71). An overnight culture was inoculated into 1 liter of LB medium, and when this culture reached an optical density at 600 nm (OD_600_) of 0.5, expression was induced with 1 mM isopropyl-β-d-1-thiogalactopyranoside (IPTG) for 16 h at 25°C. The culture was then spun at 5,000 × *g* for 10 min. The cultured medium was precipitated with 50% saturated ammonium sulfate at 4°C overnight and dialyzed against PBS overnight. Protein in the periplasm was extracted as previously described (72, 73). In brief, the cell pellet was washed with ice-cold 20% sucrose plus 30 mM Tris (pH 8) plus 1 mM EDTA, and then the periplasm was extracted with ice-cold 5 mM MgSO_4_. The periplasmic fraction was then dialyzed against PBS at 4°C overnight. Small-scale protein production was done initially to determine that rCLP ECD was abundant in both the culture medium and periplasm. As a result, the medium fraction and the periplasmic fraction were combined and loaded onto a HisPur nickel-nitrilotriacetic acid (Ni-NTA) spin column (Thermo Fisher Scientific). Purified rCLP ECD was dialyzed into PBS, and the protein concentration was determined by the Pierce BCA protein assay (Thermo Fisher Scientific).

### ITC.

Interactions of the CLPs with calcium chloride were monitored by isothermal titration calorimetry (ITC)-based experiments using an iTC200 instrument (MicroCal/GE Healthcare, Piscataway, NJ). All the protein samples were dialyzed in a buffer containing 20 mM Tris-HCl and 50 mM NaCl (pH 7.5). For ITC measurement, the sample cell (cell volume of 0.201 ml) was filled with the CLP variant (14.0 μM), and the reference cell was filled with the same buffer the protein samples were dialyzed in. The CLP variant was titrated with calcium chloride (1 mM) using the following protocol: an initial 0.2-μl injection, followed by 15 injections of 2 μl each with an interval of 3 min. The titration was carried out with constant stirring at 750 rpm at 25°C. The binding isotherm profile was obtained excluding the initial data point. The data were fitted using Origin 7 software. The *K_d_* (dissociation constant) was determined from 1/*K_a_* (where *K_a_* is binding constant).

### T. vaginalis attachment to Ects and attachment with rCLP ECD competition.

Attachment of T. vaginalis to Ects was performed as described previously (10). Briefly, 5 × 10^4^ CellTracker blue CMAC (7-amino-4-chloromethylcoumarin; Thermo Fisher Scientific)-labeled T. vaginalis organisms were incubated with confluent Ects for 30 min, and the coverslips were fixed in 4% formaldehyde in PBS and mounted on slides using Mowiol (Calbiochem). Fifteen images of each coverslip were acquired using an Axioscope 2 epifluorescence microscope (Zeiss), and cell counts were quantified using Zen lite (Zeiss) and ImageJ software (74).

Attachment assays that included rCLP ECD to compete for Ect binding were performed using WT, nontransfected RU393 parasites, and the difference in the procedure was addition of 0.25 μg, 1 μg, or 4 μg of rCLP ECD or 4 μg of BSA as a negative control to Ects for 30 min prior to parasite addition. Medium was then removed and replenished with new medium containing CellTracker blue-labeled parasites.

### Parasite clumping assay.

The parasite clumping assay was done as described previously (20), with modifications indicated below. A total of 5 × 10^5^ of CellTracker blue CMAC (Thermo Fisher Scientific)-prelabeled T. vaginalis organisms were plated at 1 × 10^6^/ml on glass coverslips covered with confluent Ects or coverslips alone and incubated in complete keratinocyte-SFM (serum-free media) with no CaCl_2_ or 1 mM CaCl_2_ for 30 min. Parasites were then fixed in 4% formaldehyde in PBS and mounted on slides using Mowiol (Calbiochem). Fifteen images of each coverslip were acquired using an Axioscope 2 epifluorescence microscope (Zeiss) and analyzed using Zen lite software. A clump is defined as an aggregate of 10 or more parasites. To calculate clumping fold changes, EV − Ca + host was set as 1-fold, so CLP − Ca would be 5.95-fold and EV − Ca would be 0.025-fold; thus, CLP − Ca/EV − Ca = a 238-fold increase. CLP − Ca + host is 7.47-fold and EV − Ca + host is 1-fold; thus, CLP − Ca + host/EV − Ca + host = a 7.47-fold increase.

### T. vaginalis-induced cytotoxicity of ectocervical cells.

Cytotoxicity of Ects was measured as described previously (10). The only modification was that 3 × 10^5^ parasites were added to confluent Ects and incubated for 4 h.

10.1128/mBio.00720-19.4 FIG S4 Mutational analysis of the rest of predicted Ca^2+^-binding sites in CLP. Phyre2 and SuSPect analyses of the other 3 predicted Ca^2+^-binding sites (from Fig. 1C). See Fig. 4A for the Ca^2+^-binding sites most sensitive to mutation. The height and color of the bars shown in the key indicate the predicted functional impact of mutating the aspartate residue to the amino acids shown at the bottom of the histogram. Long red bars in the histogram indicate that introduction of that particular amino acid would lead to the greatest phenotypic change, while short blue bars have the smallest predicted phenotypic effect. Download FIG S4, TIF file, 3.0 MB. Copyright © 2019 Chen et al. 2019 Chen et al. This content is distributed under the terms of the Creative Commons Attribution 4.0 International license.

10.1128/mBio.00720-19.5 FIG S5 T. vaginalis surface staining with a pan-cadherin antibody increases after parasite contact with Ects. RU393 parasites were exposed to Ects for 4 h. Cells were stained with an anti-pan cadherin antibody (red) without cell permeabilization to detect surface labeling. Nuclear staining with Hoechst dye is shown in blue. Tv, T. vaginalis parasites. Results are representative of those from two independent experiments with more than 100 cells viewed. Scale bar = 10 μm. Download FIG S5, TIF file, 4.5 MB. Copyright © 2019 Chen et al. 2019 Chen et al. This content is distributed under the terms of the Creative Commons Attribution 4.0 International license.
