# Supplementary figures and images for: A Novel Cadherin-like Protein Mediates Adherence to and Killing of Host Cells by the Parasite Trichomonas vaginalis
Source: mBio. 2019 May 14;10(3):e00720-19. doi: 10.1128/mBio.00720-19 (PMC6520450; doi:10.1128/mBio.00720-19)

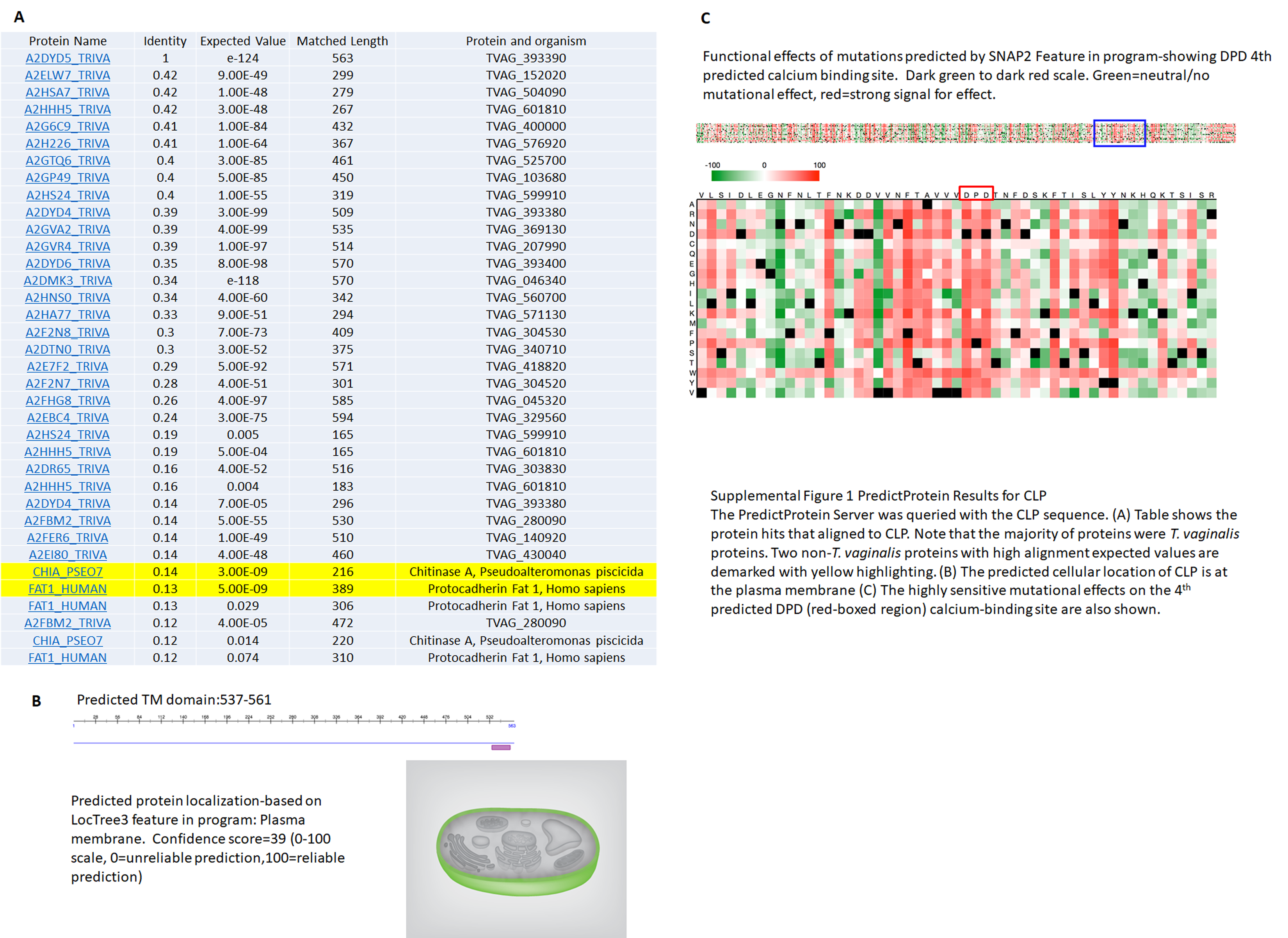

Supplement: FIG S1 [file mBio.00720-19-sf001.tif]

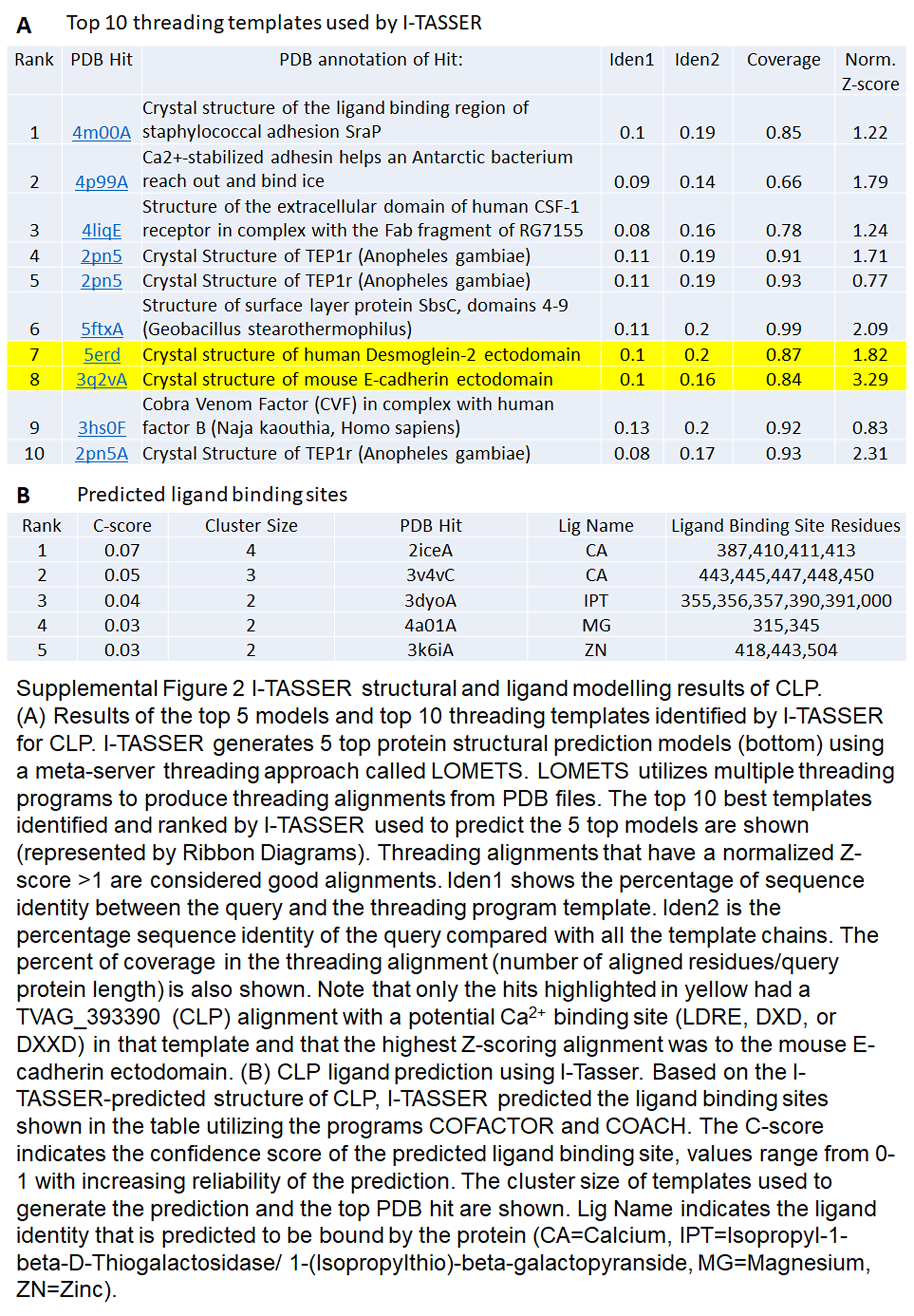

Supplement: FIG S2 [file mBio.00720-19-sf002.tif]

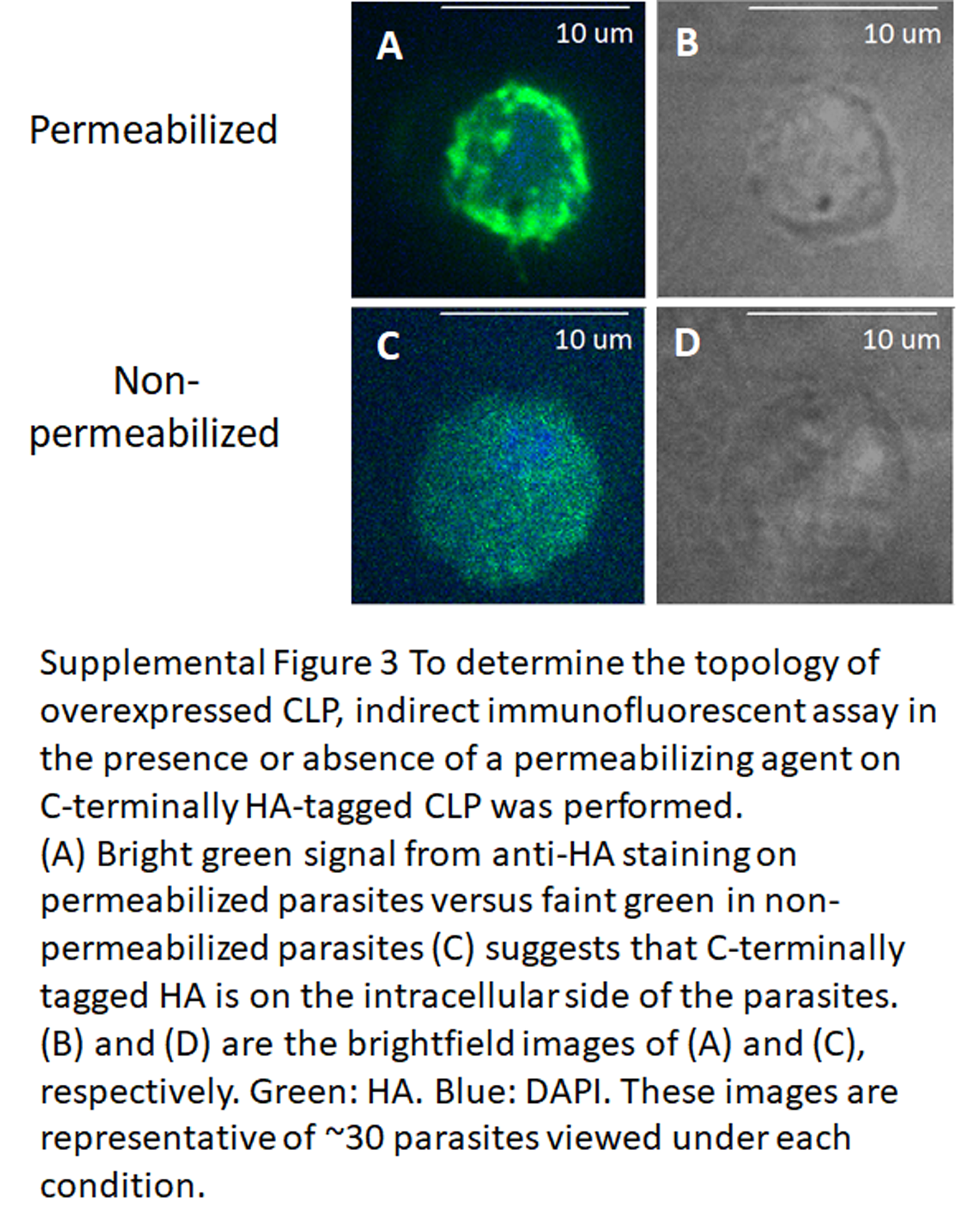

Supplement: FIG S3 [file mBio.00720-19-sf003.tif]

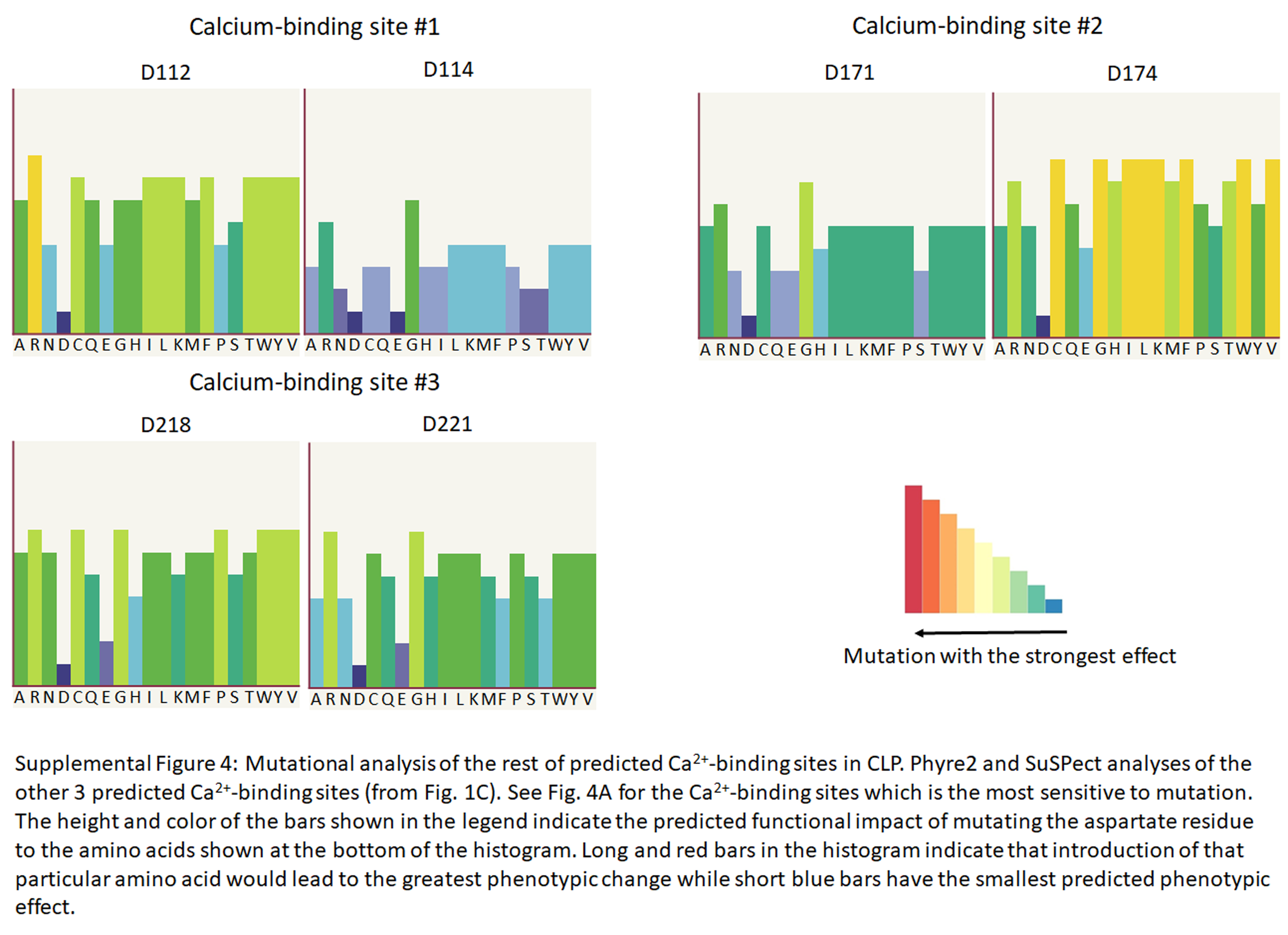

Supplement: FIG S4 [file mBio.00720-19-sf004.tif]

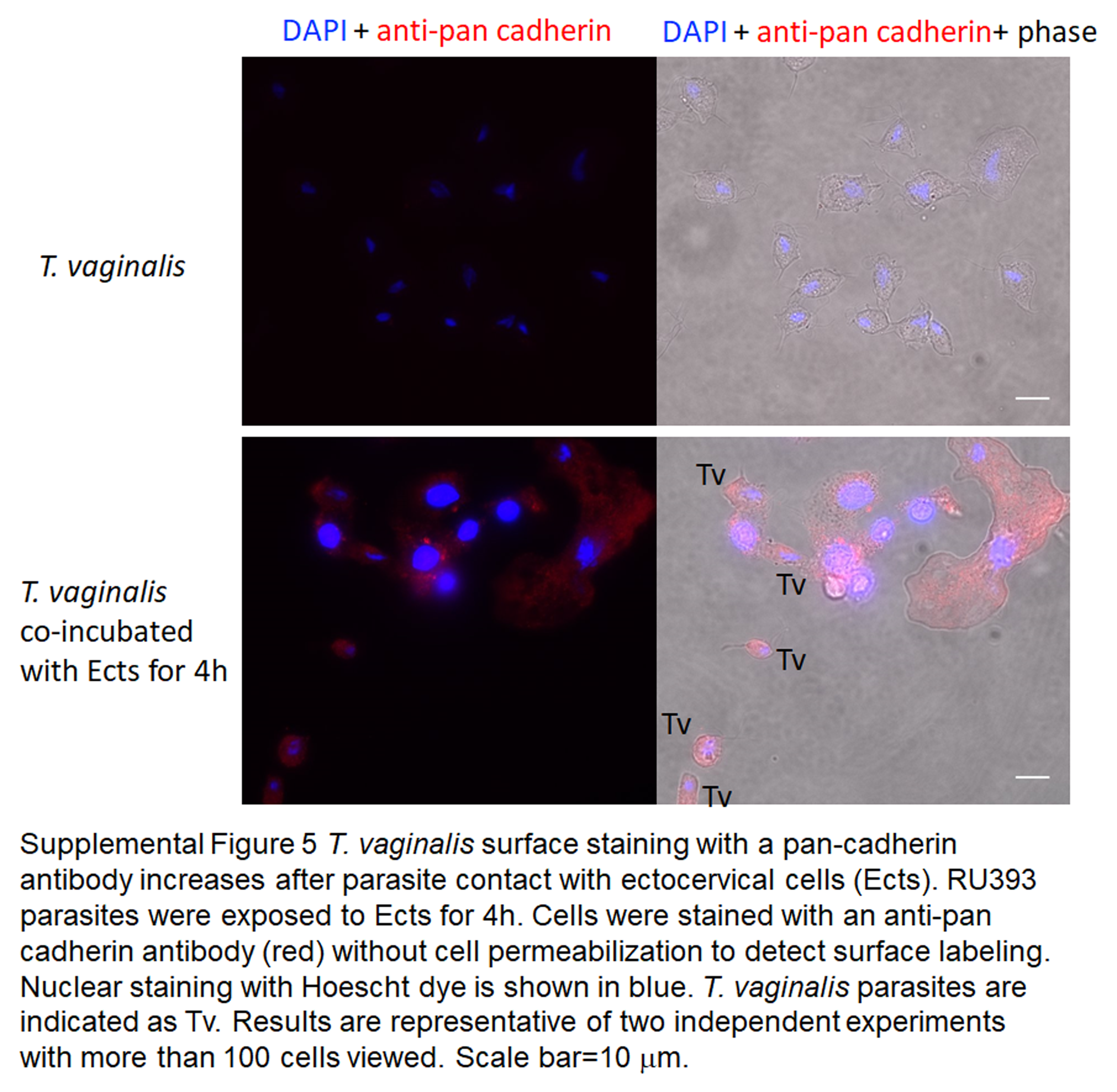

Supplement: FIG S5 [file mBio.00720-19-sf005.tif]
